# Functional Requirements for Medical Data Integration into Knowledge Management Environments: Requirements Elicitation Approach Based on Systematic Literature Analysis

**DOI:** 10.2196/41344

**Published:** 2023-02-09

**Authors:** Benjamin Kinast, Hannes Ulrich, Björn Bergh, Björn Schreiweis

**Affiliations:** 1 Institute for Medical Informatics and Statistics Kiel University and University Hospital Schleswig-Holstein Kiel Germany

**Keywords:** data integration, requirements engineering, requirements, knowledge management, software engineering

## Abstract

**Background:**

In patient care, data are historically generated and stored in heterogeneous databases that are domain specific and often noninteroperable or isolated. As the amount of health data increases, the number of isolated data silos is also expected to grow, limiting the accessibility of the collected data. Medical informatics is developing ways to move from siloed data to a more harmonized arrangement in information architectures. This paradigm shift will allow future research to integrate medical data at various levels and from various sources. Currently, comprehensive requirements engineering is working on data integration projects in both patient care– and research-oriented contexts, and it is significantly contributing to the success of such projects. In addition to various stakeholder-based methods, document-based requirement elicitation is a valid method for improving the scope and quality of requirements.

**Objective:**

Our main objective was to provide a general catalog of functional requirements for integrating medical data into knowledge management environments. We aimed to identify where integration projects intersect to derive consistent and representative functional requirements from the literature. On the basis of these findings, we identified which functional requirements for data integration exist in the literature and thus provide a general catalog of requirements.

**Methods:**

This work began by conducting a literature-based requirement elicitation based on a broad requirement engineering approach. Thus, in the first step, we performed a web-based systematic literature review to identify published articles that dealt with the requirements for medical data integration. We identified and analyzed the available literature by applying the PRISMA (Preferred Reporting Items for Systematic Reviews and Meta-Analyses) guidelines. In the second step, we screened the results for functional requirements using the requirements engineering method of document analysis and derived the requirements into a uniform requirement syntax. Finally, we classified the elicited requirements into a category scheme that represents the data life cycle.

**Results:**

Our 2-step requirements elicitation approach yielded 821 articles, of which 61 (7.4%) were included in the requirement elicitation process. There, we identified 220 requirements, which were covered by 314 references. We assigned the requirements to different data life cycle categories as follows: 25% (55/220) to data acquisition, 35.9% (79/220) to data processing, 12.7% (28/220) to data storage, 9.1% (20/220) to data analysis, 6.4% (14/220) to metadata management, 2.3% (5/220) to data lineage, 3.2% (7/220) to data traceability, and 5.5% (12/220) to data security.

**Conclusions:**

The aim of this study was to present a cross-section of functional data integration–related requirements defined in the literature by other researchers. The aim was achieved with 220 distinct requirements from 61 publications. We concluded that scientific publications are, in principle, a reliable source of information for functional requirements with respect to medical data integration. Finally, we provide a broad catalog to support other scientists in the requirement elicitation phase.

## Introduction

### Background

In patient care, data are historically generated and stored in heterogeneous data models and formats. These often result in domain specific, noninteroperable, or isolated applications and databases (data silos) [1]. As recent big data studies predict, the worldwide amount of health care data continues to grow at a high speed, becoming extremely large and complex, with isolated data silos becoming extremely large and complex and having both unstructured and structured data. To counteract isolated data storage (DS), there is continuing development in medical informatics to shift from siloed data to a more harmonized arrangement in information architectures via manifold data integration projects [2].

Haux et al [3] described data integration as a “condition of an information system in which each data item needs to be recorded, changed, deleted, or otherwise edited just once, even if it is used in several application components or contexts.” Haux et al [3] further defined data integration as “a prerequisite for the multiple usability of data*.”* Data accessibility for multiple uses supports a broad spectrum of clinical practice and enhances medical research, forming a major step toward learning about health systems. To integrate medical data at many levels and from various sources, future research will rely on this paradigm shift. Frequently addressed sources are electronic medical records and electronic health records (EHRs), laboratory information systems, Picture Archiving and Communication Systems, and mobile health devices [4-7].

One of the main challenges the heterogeneity of these siloed data poses is caused by organizational and technical challenges [8-10]. These challenges are tackled in various local, national, and international projects in both patient care– and research-oriented contexts [11,12].

A prominent project for comprehensive data integration at the national level is the German Medical Informatics Initiative. The initiative’s major goal is to develop an infrastructure to integrate clinical data from patient care and medical research in German University Hospitals [13] using different technological and organizational approaches. The approaches differ between the participating university hospitals and the respective consortia [14-17]. These hospitals integrate medical data into data integration centers by pursuing use case–driven integration approaches to support research.

University Hospital Schleswig-Holstein is a part of the Medical Informatics Initiative. Our integration approach is more holistic and sustainable, as it includes preparing and providing data directly for patient care. In doing so, we are contributing to the paradigm shift and decreasing the boundaries between patient care and research [18]. It is based on Haux et al’s [3] assumption that IT systems or platforms need to provide “the right information [...] and the right knowledge [...] at the right time in the right place for the right people in the right form so that these people can make the right decisions” (*information and knowledge logistics*). We refer to such a generic platform as a knowledge management platform that incorporates, manages, and provides both patient care and research data.

Even though the framework conditions and goals of general integration projects differ, and no 2 integration projects are alike at the onset, they follow a more or less formalized process of specifying the requirements for elicitation. Therefore, these projects involve different stakeholders as the basis of each software development project [19]. Careful elicitation of requirements guarantees the subsequent quality of a software system. Various industry-related studies have shown that unclear, ambiguous, or incomplete requirements are the main cause of failing the objectives of software projects [20,21]. Giodarno [22] points out that data integration is not considered traditional application development as “[...] the data integration development life cycle places the bulk of the effort in the design phases.” As conventional software development often starts from scratch, data integration projects must analyze the already existing system environment in more detail and incorporate it into the conceptual design. Consequently, this highlights the importance of comprehensive requirement elicitation in data integration projects.

As careful elicitation of the requirements guarantees the subsequent quality of a software system, we raised the question of how we could achieve the highest possible requirements’ quality to reduce subsequent risks of one-sided, imprecise, or misleading requirements while developing a data integration environment [20,21]. This further led to the question of which methods, beyond established stakeholder-based methods, such as interviews, questionnaires, brainstorming sessions, agile methods, or prototyping, could support the elicitation process [23]. To broaden this spectrum, the approach of learning from the stakeholder requirements of other projects seemed promising.

Therefore, we decided to pursue a broad requirements engineering approach by conducting a literature-based requirement elicitation. Nonfunctional requirements are usually subject to national legal frameworks or other individual qualitative requirements of stakeholders ensuring system performance, availability, or reliability, whereas functional requirements, describe the interaction of a system with its environment [24]. In our opinion, functional requirements are more likely to be transferable to other projects, as they are determined by the technical framework of an integration environment. Therefore, we focused our method of choice on functional requirement aspects.

### Objectives

Our main objective was to provide a general catalog of functional requirements for the integration of medical data into knowledge management environments. In the first step, we aimed to identify which functional data integration requirements can be found in the literature. In the next step, we wanted to identify the intersections between these requirements to derive consistent and representative functional requirements.

To subdivide requirements in a clear and selective manner, we applied a category scheme and evaluated its benefits in the context of our requirement elicitation project.

## Methods

### Overview

The methodology of this study was based on a 2-step approach conducting a requirement elicitation based on a systematic literature review. To compose requirements into a uniform requirement syntax, we screened the requirements results using the requirements engineering method of document analysis [24,25].

### Systematic Literature Review

In the first step, we performed a web-based systematic literature review to identify published articles that dealt with the requirements for medical data integration. To ensure the proper organization and transparency of our results, we identified and analyzed the available literature by applying the PRISMA (Preferred Reporting Items for Systematic Reviews and Meta-Analyses) guidelines.

### Requirements Engineering

In the second step, we applied the qualitative document-based requirement elicitation approach to scientific literature [24,25]. To identify functional requirements, we screened the articles that matched our inclusion criteria (*Inclusion and Exclusion Criteria* section). In this process, we considered both the implicit and explicit requirements. The identified requirements were transferred to a category scheme, which has been described in detail in the section *Category Scheme*.

### Search Strategy

In December 2020, we conducted a large-scale, comprehensive, and systematic web-based search focusing on English-language articles published before November 30, 2020. In PubMed, CINAHL, Cochrane Library, Scopus, and Web of Science, we first searched for the keywords “Requirements” and “Data Integration.” In the Web of Science database, we further limited the search results to include only “Medical Informatics” and “Healthcare science services” and in the Scopus database, to the broad domains of “Medicine” and “Computer Science.” The exact queries sorted by database are listed in Table 1.

**Table 1:** Queries.

Database	Query	Additional filters
PubMed	“((requirements[Title/Abstract]) AND (data integration[Title/Abstract])) AND ((“1900/01/01”[Date - Publication] : “2020/11/30”[Date - Publication]))”	N/A^a^
CINAHL	“AB Requirements AND AB data integration”	Limitations - Publication date:19000101-20201130; In English
Cochrane Library	“(requirements):Title Abstract Keyword AND (data integration): Title Abstract Keyword”	“(Word variations have been searched)” with Cochrane Library publication date Between Jan 1990 and Nov 2020, in Cochrane Reviews
Web of Science	“((TS=(requirements and data integration) OR AB = (requirements and data integration)))” AND LANGUAGE: (English)	Refined by: WEB OF SCIENCE CATEGORIES: (MEDICAL INFORMATICS OR HEALTH CARE SCIENCES SERVICES )Indexes=SCI-EXPANDED, SSCI, A&HCI, ESCI Timespan=1945-2020
Scopus	SUBJAREA (comp) SUBJAREA (medi) (TITLE(requirements) OR ABS (requirements) AND TITLE (data AND integration) OR ABS (data AND integration)) AND (EXCLUDE(PUBYEAR, 2021)) AND (LIMIT-TO(LANGUAGE, “English”)) AND (EXCLUDE(DOCTYPE, “ch”))	N/A

^a^N/A: not applicable.

### Inclusion and Exclusion Criteria

We included articles that met the following criteria: (1) full-text publications in the context of medical data integration (2) mentioning functional data integration requirements. For the purpose of this requirements elicitation approach, we defined functional requirements by following ISO 29148. According to ISO 29148, the functional requirements describe the system or system element functions or tasks to be performed by the system. Thus, we considered all other requirements to be nonfunctional [26]. To create a uniform understanding of data integration, we used the definition of Haux et al [3]. Following our strategy, we did not exclude any specific type of publication per se, but focused on publications that addressed a theoretical or practical medical data integration approach with medical data. Therefore, we excluded publications without reference to medical data integration and those focusing on nonfunctional requirements.

### Selection and Data Extraction

We retrieved the results of each search performed in the databases using Citavi 5 (version 5.7.1.0, Swiss Academic Software GmbH). First, we removed all duplicates. Second, the corresponding author (BK) identified relevant articles by screening all titles and abstracts based on our selection criteria; records that clearly did not meet the eligibility criteria were excluded. Subsequently, the corresponding author—an experienced reviewer in the field of requirements engineering—assessed all eligible and freely available full-text publications. While conducting the full text–based requirements elicitation, we identified potentially relevant references in the first-level results based on the context for further backward reference tracking.

### Category Scheme

To structure the technical dimensions of the functional requirements, we adapted the *Data Lake Life Cycle* by John and Misra [27] as the basis for our category scheme. It describes the various stages of data as they live within knowledge management systems (ie, data lake and data warehouse), namely *data acquisition* (DA)*, data processing* (DP)*, data analysis* (DAS)*,* DS*, metadata management* (MDM)*, data traceability* (DT)*, data lineage* (DL)*,* and *data security* (DSC) [27]. To clearly delineate the categories and establish a uniform understanding, we used the literature-based definitions presented in Textbox 1.

**Definition of categories.**

- Data acquisition
  The process of gathering, filtering, and cleaning data before they are put in a data warehouse or any other storage solution in which data analysis can be carried out [28].
- Data processing
  The process of collecting and manipulating items of data to produce meaningful information [29].
- Data analysis
  The process of exploring, transforming, and modeling data with the goal of synthesizing, highlighting, and extracting relevant information [28].
- Data storage
  The process of how hardware or software holds, deletes, backups, organizes, and secures information. This includes keeping data in temporary or permanent storage [30].
- Data lineage
  The process of changes during the data life cycle, which includes the data’s origins and where they moves over time [27].
- Data traceability
  The process of verifying the history, location, or application of an item by means of documented recorded identification [27].
- Data security
  The process of protecting digital information from unauthorized access, corruption, or theft throughout its entire life cycle [31].
- Metadata management
  The process of generating and managing metadata by (1) minimizing the efforts for the development and administration of a knowledge management system (eg, data warehouse) and (2) to improve the extraction of information from the metadata [32].

### Requirement Elicitation

We scanned the resulting literature for both implicit and explicit functional requirements. Next, we extracted text passages containing direct or indirect requirements or both and transferred them to the category scheme (Textbox 1) for further evaluation. The subsequent content evaluation involved four steps: (1) an expert on requirement elicitation extrapolated text passages describing functional requirements from the articles; (2) the expert evaluated those passages, assigned a category (and if helpful, a subcategory for a better overview), and derived requirements according to the specified syntax; (3) he then discussed and evaluated the derived requirements with a senior in the field of medical data integration; and (4) once they reached a consensus, they pooled matching requirements from different publications in a mind map to form uniform requirements wherever possible.

In requirements engineering, requirements are usually assigned different commitment levels. A distinction is made between excitement factors, satisfiers, and dissatisfiers, which are differentiated syntactically when writing a requirement, for example, “the system should,” “the system must,” and “the system must not.” In the context of our requirement elicitation, we selected satisfiers (“must”) [24]. We applied the requirement syntax in accordance with ISO 29148: [Subject] [Action] [Constraint of Action] [26], for example, “the system must integrate data.” If the requirements contain >1 action condition, either “[...] all of the following: * Option A * Option B * Option C.” is applied as a logical “AND” or “[...] one of the following: * Option A * Option B * Option C.” is applied as a logical “OR” and “at least one of the following: * Option A * Option B * Option C.” as a logical “XOR.” Requirements that contain examples from the reference literature, such as a source system to be integrated or a standard to be adopted, are annotated with a list of examples in the notes.

## Results

### Overview

**Figure 1:**
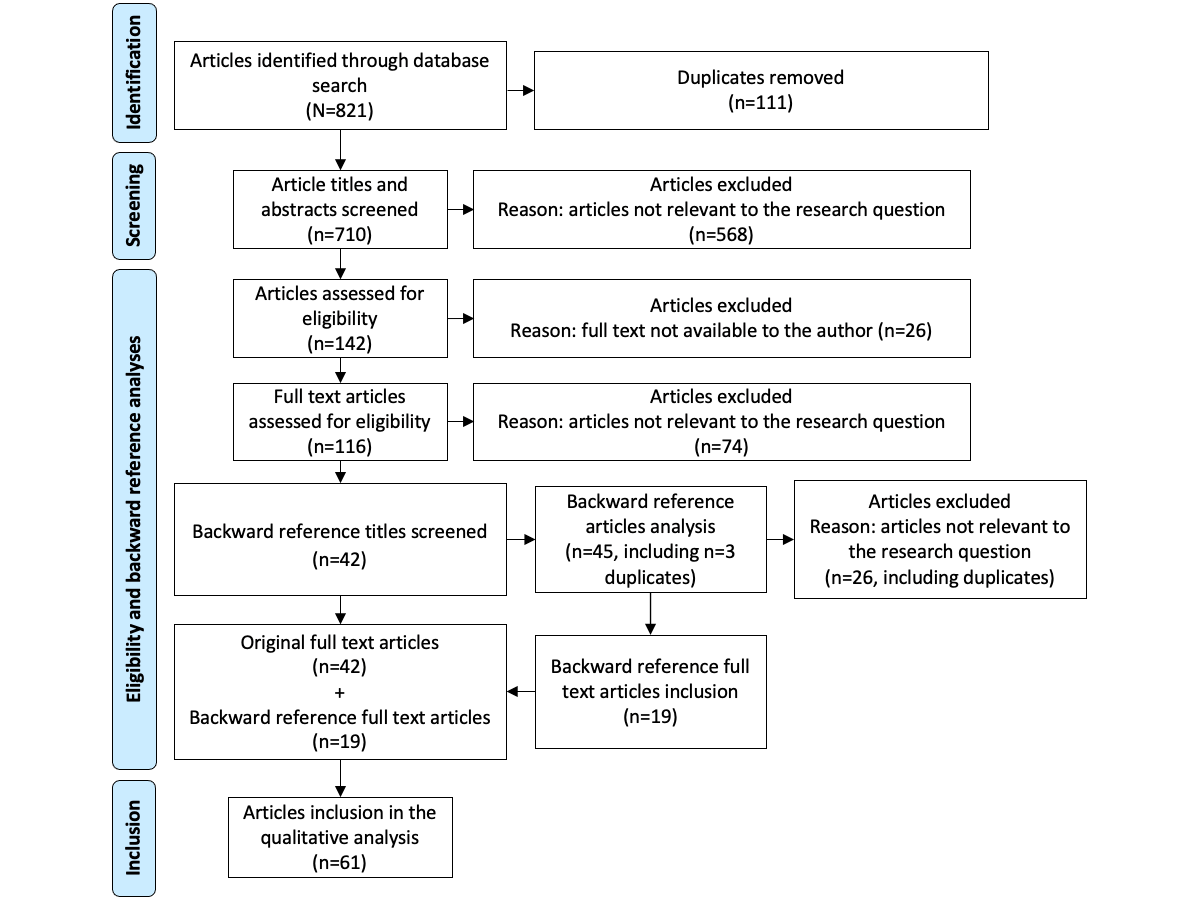
PRISMA (Preferred Reporting Items for Systematic Reviews and Meta-Analyses) flow diagram of the literature screening process.

We identified 821 articles through a systematic literature search. After removing duplicates, we included a total of 86.5% (710/821) of articles for a detailed title and abstract screening. This led to the exclusion of 80% (568/710) of articles that were considered irrelevant to our research scope. In the 20% (142/568) of remaining articles, we performed a full-text evaluation, resulting in the inclusion of 29.6% (42/142) of articles. We then performed backward reference tracking and included 19 additional articles. Finally, 61 articles formed the basis of the systematic review. An illustration of the selection process can be found as PRISMA flow diagram in Figure 1.

We identified a total of 314 requirements in the literature. These were then merged into 220 requirements.

Finally, we assigned 25.5% (56/220) of requirements (35/61, 57% publications) to the DA category, 35.9% (79/220) of requirements (49/61, 80% publications) to DP, 12.7% (28/220) of requirements (19/61, 31% publications) to DS, 9.1% (20/220) of requirements (14/61, 23% publications) to DAS, 6.4% (14/220) of requirements (15/61, 25% publications) to MDM, 2.3% (5/220) of requirements (5/61, 8% publications) to DL, 3.2% (7/220) of requirements (5/61, 8% publications) to DT, and 5.5% (12/220) of requirements (11/61, 18% publications) to the category DSC.

Given the total number of requirements evaluated, this paper presents only a list of the selected requirements (by occurrence in individual publications) per category. A complete overview of all the requirements and related publications can be found in Multimedia Appendix 1.

### Data Acquisition

We assigned 25.5% (55/220) of requirements from 57% (35/61) of publications to the DA category. Owing to the total number of requirements, we limited ourselves to presenting the requirements with 3 to 6 articles. A complete list of requirements can be found in Multimedia Appendix 1 requirements. The most cited requirement is *DA-01* with 5 articles [11,33-36] and 6 further subrequirements, *DA-01-1* to *DA-01-6* (Multimedia Appendix 1—requirements). The most referenced DA requirements are listed in Table 2. The first one, *DA-01*, states that data are integrated from different heterogeneous source systems. In this sense, the 5 studies [11,33-37] mention different source systems, which include Health Care Information Systems in general, Laboratory Information Management Systems, and Clinical Trial Management Systems. These integration projects span various medical domains, including clinical trials in general, systems biology, and OMICS. *DA-02* describes (4 studies [38-41]) the necessity to acquire structured data based on general interoperability standards from health IT such as Institute of Electrical and Electronics Engineers or Bluetooth low energy as well as standards from the health care domain such as Continua Health Alliance, Health Level 7 Fast Health Care Interoperability Resources, or Digital Imaging and Communications in Medicine (DICOM). *DA-01-1* (3 articles [42-44]) extends *DA-01* to include different data from electronic medical records or EHRs containing standard data sets about patients’ demographics and more specific data sets about vital signs, laboratory tests, medication data, diagnoses, and procedures. *DA-03* was derived from 3 publications [36,45,46], each of which describes the integration of various multimedia data. These include waveform, sound, and images from domains such as medical imaging (radiology) and biomedical instrumentation [36,45,46]. The final requirement in this list is *DA-05* (3 articles [44,47,48]). *DA-05* points out the need for an abstraction layer for acquiring both data and metadata from heterogeneous sources such as Patient Management Systems.

**Table 2:** Top 6 data acquisition (DA) requirements.

Requirement ID	Requirement	Note	Source	Articles, n	Parent
DA-01	The system must acquire data from different heterogeneous source systems.	Possible heterogeneous source systems are LIMS^a^, HIS^b^, and CTMS^c^.	Ethier et al [11], Lelong et al [33], Tahar et al [34], Tsiknakis et al [35], and Gupta et al [36]	5	N/A^d^
DA-02	The system must acquire structured data based on interoperability standards from IT and the health care domain.	Possible interoperability standards are HL7 FHIR^e^, DICOM^f^, IEEE^g^, Continua Health Alliance, and BTLE^h^.	Urbauer et al [38], Katehakis et al [39], Firnkorn et al [40], and Haak et al [41]	4	N/A
DA-01-1	The system must acquire at least one of the following: *EHR^i^ data, *EMR^j^ data.	Possible EHR or EMR data are demographics, vital signs, laboratory tests, medication, diagnoses, and procedures.	Mo et al [42], Gaff et al [43], and Bahls et al [44]	3	DA-01
DA-03	The system must acquire multimedia data from various domains.	Possible multimedia data types are waveform, sound, and images and possible domains are medical imaging and biomedical instrumentation.	Gupta et al [36], Rajasekaran et al [45], and Katehakis et al [46]	3	N/A
DA-05	The system must provide an abstraction layer to acquire (meta) data from heterogeneous sources.	Possible heterogeneous sources are PMS^k^.	Bahls et al [44], Ozyurt et al [47], and Oliveira et al [48]	3	N/A

^a^LIMS: Laboratory Information Management System.

^b^HIS: Health Care Information System.

^c^CTMS: Clinical Trial Management Systems.

^d^N/A: not applicable.

^e^HL7 FHIR: Health Level 7 Fast Health Care Interoperability Resources.

^f^DICOM: Digital Imaging and Communications in Medicine.

^g^IEEE: Institute of Electrical and Electronics Engineers.

^h^BTLE: Bluetooth low energy.

^i^EHR: electronic health record.

^j^EMR: electronic medical record.

^k^PMS: Patient Management Systems.

### Data Processing

We identified 35.9% (79/220) of requirements in 80% (49/61) of publications for the category DP. Thus, DP turned out to be the largest category (Table 3). Owing to the number of requirements, we limited ourselves to presenting the requirements with 4 to 18 articles. The remaining 92% (73/80) of requirements can be found in Multimedia Appendix 1 requirements. The first requirement, *DP-01*, was mentioned in 18 publications [10,12,34,39,40,46,48-59] and, therefore, the most cited requirement of this study. It describes the need to map data to international terminology standards such as SNOMED-CT, LOINC, ICD, ATC, ICD-O, CPT, and RxNorm as part of processing. Subordinated to this processing requirement is *DP-01-1* (5 articles [12,42,46,55,60]), which requires mapping between not only international standards but also local terminologies and their national or international standardized counterparts. Following this, *DP-01-2* (4 articles [35,56,61,62]) specifies the need to map data to standardized semantics, vocabularies, and ontologies in general when processing data. *DP-03* (4 articles [47,63-65]) requests that during the integration, (semi)structured data be processed into common exchange formats such as XML, CSV, or JSON. *DP-02* (6 articles [12,42,57,58,66,67]) describes the need for the application of natural language processing (NLP) methods to integrate free-text data. To conclude, *DP-04* (4 articles [68-71]) requires standardized rules or processes when it comes to processing data from various sources.

**Table 3:** Data processing (DP) requirements.

Requirement ID	Requirement	Note	Source	Articles, n	Parent
DP-01	The system must process data by mapping it to multiple terminology standards.	Possible terminology standards are SNOMED-CT^a^, LOINC^b^, ICD^c^, ATC^d^, IDC-O^e^, CPT^f^, and RxNorm.	Sujansky [10], Chute et al [12], Tahar et al [34], Katehakis et al [39], Firnkorn et al [40], Katehakis et al [46], Oliveira et al [48], Marschollek [49], Bestek and Stanimirovic [50], Ayatollahi et al [51], Daniel et al [52], Denecke et al [53], Deserno et al [54], Ethier et al [55], Ethier et al [56], Rance et al [57], Hall et al [58], and Lowe et al [59]	18	N/A^g^
DP-02	The system must process unstructured data using NLP^h^ methods.	Possible NLP methods are text mining, identification of targeted document types, section location, concept identification, negation, and context filtering.	Chute et al [12], Mo et al [42], Rance et al [57], Hall et al [58], Bouzillé et al [66], and Botsis et al [67]	6	N/A
DP-01-1	The system must process data by mapping between local terminologies to at least one of the following: *national terminology standards, *international terminology standards.	Possible terminology standards are ICD-10^i^, ATC, SNOMED-CT, LOINC and PathLex^j^.	Chute et al [12], Mo et al [42], Katehakis et al [46], Ethier et al [55], and Doods et al [60]	5	DP-01
DP-01-2	The system must process data by mapping it to all the following: *standardized semantics, *vocabularies, *ontologies.	N/A	Tsiknakis et al [35], Ethier et al [56], Miles et al [61], and Martone et al [62]	4	DP-01
DP-03	The system must process (semi)structured data to a common exchange format.	Possible formats are XML, CSV, and JSON.	Ozyurt et al [47], Wong [63], Hamid et al [64], and Karasavvas et al [65]	4	N/A
DP-04	The system must process data from various sources based on at least one of the following: *standardized rules, *standardized process.	N/A	Ozaydin et al [68], Hanss et al [69], Welch et al [70], and Ganzinger et al [71]	4	N/A

^a^SNOMED-CT: Systematized Nomenclature of Medicine.

^b^LOINC: Logical Observation Identifiers Names and Codes.

^c^ICD: International Statistical Classification of Diseases and Related Health Problems.

^d^ATC: Anatomical Therapeutic Chemical.

^e^IDC-O: International Classification of Diseases for Oncology.

^f^CPT: Current Procedural Terminology.

^g^N/A: not applicable.

^h^NLP: natural language processing.

^i^ICD-10: International Statistical Classification of Diseases and Related Health Problems, 10^th^ Revision.

^j^PathLex: Anatomic Pathology Lexicon.

### Data Storage

As a result of the search for DS requirements, we derived a total of 12.7% (28/220) of requirements from 31% (19/61) of publications. Owing to the number of requirements, we limit ourselves to presenting the requirements with 2 articles; the remaining 79% (22/28) of requirements can be found in Multimedia Appendix 1. *DS-01* is from 4 articles [12,59,63,72] and thus the most cited one. In addition, a 1-child requirement was assigned. *DS-01* states that data must be stored according to a multidimensional data model, such as the Entity-Attribute-Value. The next requirement, *DS-02-1,* is in a parent-child relationship with *DS-02*. *DS-02-1* (2 articles [58,73]) specializes the request from its parent requirement and describes the need to store biomedical data decentralized from clinical EHR data, for instance, locally per site. Furthermore, the requirement *DS-03* (2 articles [66,74]) points out that in addition to processed data, the original data must be stored as well. *DS-04* (2 articles [74,75]) expresses the need to make data retrievable by queries after storing them. Possible query technologies mentioned are SQL queries and Xqueries. All the aforementioned requirements are listed in Table 4.

**Table 4:** Data storage (DS) requirements.

Requirement ID	Requirement	Note	Source	Articles, n	Parent
DS-01	The system must store data according to a multidimensional data model.	A possible multidimensional data model is EAV^a^.	Chute et al [12], Lowe et al [59], Wong [63], and Hackl and Ammenwerth [72]	4	N/A^b^
DS-02-1	The system must store biomedical data decentralized from clinical EHR^c^ data.	A possible storage location is each local per site.	Hall et al [58] and Teodoro et al [73]	2	DS-02
DS-03	The system must store the original data.	N/A	Karasavvas et al [66] and Ohmann and Kuchinke [74]	2	N/A
DS-04	The system must store the data in such a way that they can be retrieved via queries.	Possible technologies for queries are SQL queries and Xqueries.	Ohmann and Kuchinke [74] and Duftschmid et al [75]	2	N/A

^a^EAV: Entity-Attribute-Value.

^b^N/A: not applicable.

^c^EHR: electronic health record.

### Data Analysis

Next is the category of DAS, with a total of 9.1% (20/220) of requirements derived from 23% (14/61) of publications. In this category, we show a selection of 5 requirements with 1 to 2 articles (Table 5). The remaining 75% (15/61) of requirements can be found in Multimedia Appendix 1. *DAS-01* requests DAS for data cleansing, which is supported by 1 publication [73]. The article refers to an example of semiautomatic data cleansing. It was supplemented by 2 children, *DAS-01-1* and *DAS-01-2*, with 1 article for *DAS-01-1* [57] and 2 for *DAS-01-2* [51,57]. Rance et al [57] stated that data must be analyzed for consistency checks (*DAS-01-1*). They further expressed the need to find or report missing values (*DAS-01-2*) [57], which was also requested by Ayatollahi et al [51]. Following this, *DAS-02* (1 article) focuses on the downstream analysis of unstructured data using NLP methods [76]. *DAS-03* (1 article [72]) broadens this demand to include diverse DAS techniques such as querying, data mining, machine learning, and semantic mining [72].

**Table 5:** Data analysis (DAS) requirements.

Requirement ID	Requirement	Note	Source	Articles, n	Parent
DAS-01	The system must analyze the data for data cleansing.	A possible method for data cleansing is semiautomatic data cleansing.	Teodoro et al [73]	1	N/A^a^
DAS-01-2	The system must analyze the data to report all of the following: *missing values, *outliers.	N/A	Ayatollahi et al [51] and Rance et al [57]	2	DAS-01
DAS-01-1	The system must analyze the data for consistency checks.	N/A	Rance et al [57]	1	DAS-01
DAS-02	The system must analyze unstructured data using NLP^b^ methods.	N/A	Denecke et al [76]	1	N/A
DAS-03	The system must provide various data analysis techniques.	Possible DAS techniques are querying, data mining, machine learning, and semantic mining.	Hackl and Ammenwerth [72]	1	N/A

^a^N/A: not applicable.

^b^NLP: natural language processing.

### Data Lineage

With respect to DL, we identified 2.3% (5/220) of requirements, which are covered by 2% (1/61) of publications each, all of which are presented in Table 6. Requirement *DL-01* addresses changes in the source data, data sources, clinical context, or analyzed requirements themselves while calling for the ability to iterate the data integration process [72]. *DL-02* states the necessity to back reference and depseudonymize personal data [39]. *DL-03* requests a functionality that provides an audit trial if the source data are changed during the course of an audit [74]. In addition, Ganzinger et al [71] raised the requirement *DL-04* to provide information for identifying the location of a service providing data. To conclude, *DL-05* addresses the transfer property files and logs, which should be created when integrating data [69].

**Table 6:** Data lineage (DL) requirements.

Requirement ID	Requirement	Note	Source	Articles, n	Parent
DL-01	The system must iterate data integration if at least one of the following change: *source data, *data sources, *clinical context, *analyzed requirements.	N/A^a^	Hackl and Ammenwerth [72]	1	N/A
DL-02	The system must provide a patient identification service.	N/A	Katehakis et al [39]	1	N/A
DL-03	The system must provide an audit trail if the source data change during the course of audits.	N/A	Ohmann and Kuchinke [74]	1	N/A
DL-04	The system must provide information to identify a service’s location.	N/A	Ganzinger and Knaup [71]	1	N/A
DL-05	The system must create all of the following when integrating data: *transfer property file, *log file.	N/A	Hanss et al [69]	1	N/A

^a^N/A: not applicable.

### Data Traceability

The category DT includes 3.2% (7/220) of requirements, each taken from one of 8% (5/61) of different publications. All requirements are shown in Table 7 and Multimedia Appendix 1. *DT-01* points out that data traceability to the primary data should be guaranteed [77]. *DT-01-1* specifies this parent requirement by stating that a direct link to the original data must be provided [74]. *DT-02* states that data indexes should be consulted, allowing data to be traced to the source systems [39]. Further data tracing is requested in *DT-03*, which mentions the need for traceability using pipeline management [71]. *DT-04* supports the need for the reproducibility of queries executed on the data [12]. *DT-05* requires tracking data to both the location of the data source and associated source data documents. Finally, *DT-06* expresses the necessity to trace the correspondence between the original data and copies, including metadata [74].

**Table 7:** Data traceability (DT) requirements.

Requirement ID	Requirement	Notes	Source	Articles, n	Parent
DT-01	The system must track data to the primary data.	N/A^a^	Hackl et al [77]	1	N/A
DT-01-1	The system must provide a link to the original data.	N/A	Ohmann and Kuchinke [74]	1	DT-01
DT-02	The system must track data via the data indexes of the source systems.	N/A	Katehakis et al [39]	1	N/A
DT-03	The system must track data via pipeline management.	N/A	Ganzinger and Knaup [71]	1	N/A
DT-04	The system must track the reproducibility of query results at different points in time.	N/A	Chute et al [12]	1	N/A
DT-05	The system must track data to all of the following: *the location of sources, *associated source data documents.	N/A	Ohmann and Kuchinke [74]	1	N/A
DT-06	The system must track data to check the correspondence between the copy and the original (including metadata).	N/A	Ohmann and Kuchinke [74]	1	N/A

^a^N/A: not applicable.

### Metadata Management

The category MDM included 6.4% (14/220) of requirements elicited from 25% (15/61) of publications. The 36% (5/14) of selected requirements with 2 articles are listed in Table 8. Although *MDM-01* (2 articles [12,72]) states a broad need for an undefined MDM tool, the child requirement *MDM-01-1* (2 articles [40,78]) expresses a more specific need for a semantic metadata repository. Furthermore, support for structural metadata mappings and structural metadata transformations is requested in *MDM-02* [40,79]. In *MDM-03* (2 articles [47,80]), the general need for metadata from multiple sources is requested, and it refers to the corresponding metadata models Health Care Domain Reference Data Model and Data Tag Suite as possible examples. *MDM-05* (2 articles [39,46]) states that a registry service must be provided to assist in identifying and accessing organizations, devices, and software. The remaining 64% (9/14) of requirements can be found in Multimedia Appendix 1.

**Table 8:** Metadata management (MDM) requirements.

Requirement ID	Requirement	Notes	Source	Articles, n	Parent
MDM-01	The system must provide metadata management tools.	N/A^a^	Chute et al [12] and Hackl and Ammenwerth [72]	2	N/A
MDM-01-1	The system must store metadata into a semantic MDR^b^.	N/A	Firnkorn et al [40] and Daniel et al [78]	2	MDM-01
MDM-02	The system must support all of the following: *structural metadata mappings, *structural metadata transformations.	A possible mapping is from the local database metadata to the CDIM^c^.	Firnkorn et al [40] and Ethier et al [79]	2	N/A
MDM-03	The system must process metadata from multiple sources according to a metadata model.	Possible metadata models are DATS^d^ and HRDM^e^.	Ozyurt and Grethe [47] and Leisch et al [80]	2	N/A
MDM-05	The system must provide a registry service to identify and access all of the following: *organizations, *devices, *software.	N/A	Katehakis et al [39] and Katehakis et al [46]	2	N/A

^a^N/A: not applicable.

^b^MDR: metadata repository.

^c^CDIM: Clinical Data Integration Model.

^d^DATS: Data Tag Suite.

^e^HRDM: Health Care Domain Reference Data Model.

### Data Security

For the last category, DSC, we identified a total of 5.5% (12/220) of requirements from 18% (11/61) of publications. Table 9 presents 33% (4/12) of requirements with 3 to 6 articles. The first, *DSC-01* (6 articles [39,40,46,57,72,74]), requires that the system environment storing the data provide user authentication and authorization to ensure overall DSC. *DSC-02* (4 articles [39,46,57,69]) requests encryption and decryption for (cross-institutional) institutional data transfers. *DSC-03* (3 articles [35,45,73]) adds a legal component to DSC requirements by requesting data privacy and security in accordance with legal or ethical regulations, which may result, for example, from European data privacy regulations. The last DSC requirement to be mentioned is *DSC-04* (3 articles [54,57,69]), which requires the disconnection of medical data from identifying data using a pseudonymized ID (PID). The remaining 73% (8/14) of requirements can be found in Multimedia Appendix 1.

**Table 9:** Data security (DSC) requirements.

Requirement ID	Requirement	Notes	Source	Articles, n	Parent
DSC-01	The system must provide all of the following: *user authentication, *use authorization.	N/A^a^	Katehakis et al [39], Firnkorn et al [40], Katehakis et al [46], Rance et al [57], Hackl and Ammenwerth [72], and Ohmann and Kuchinke [74]	6	N/A
DSC-02	The system must provide encryption and decryption for (cross-institutional) data transfer.	N/A	Katehakis et al [39], Katehakis et al [46], Rance et al [57], and Hanss et al [69]	4	N/A
DSC-03	The system must provide data privacy and security in accordance with at least one of the following: legal regulations, ethical regulations.	Possible legal and ethical regulations include those that result from the European data privacy regulations.	Tsiknakis et al [35], Rajasekaran et al [45], and Teodoro et al [73]	2	N/A
DSC-04	The system must provide a PID^b^ to disconnect medical data from identifying data.	N/A	Deserno et al [54], Rance et al [57], and Hanss et al [69]	3	N/A

^a^N/A: not applicable.

^b^PID: pseudonymized ID.

## Discussion

### Principal Findings

The aim of this study was to systematically investigate functional data integration requirements, which were elicited from and published in the academic literature. The literature we analyzed stemmed from 5 highly relevant databases and covered up to 3 decades of research on data integration and the associated requirements. To keep the scope of results within a reasonable range, we searched for “requirements” in addition to “data integration.” This left out publications without mention of requirements in the title and abstract. We compensated for this limitation by performing reference tracking.

During the first phase of our systematic literature review, we identified 821 publications from the medical domain. During the second phase, we limited the publications relevant to our requirement elicitation method to 7.4% (61/821). From these 61 publications, we gathered a solid cross-section of requirements from different data integration projects in health care. With 220 identified requirements, this study represents the most comprehensive and holistic publication on the topic of requirements for data integration in the medical context.

Another distinctive feature is our adaptation of the data life cycle schema as the basic framework for this research. With 8 categories, we had a differentiated category scheme at our disposal that supported sorting and delimiting requirements at a granular level. We could assign all the requirements to a category, although the number of requirements was unevenly distributed.

This said, the DA category is characterized by a variety of requirements that have the goal of acquiring data from various source systems such as EHRs, Health Care Information Systems, and Laboratory Information Management Systems. The acquisition method, file format, and corresponding standards play superordinate roles. The requirements of the DP category largely address the transformation of acquired data into usable formats as well as the annotation of and mappings to international terminologies and code systems. In this context, interoperability aspects such as format integration and semantic and syntactic integration seem to be key factors in most requirements [55]. Overall, the results show that the requirements of these 2 categories are overrepresented and particularly discussed. This can be attributed to the fact that these categories tackle a continuing challenge of data integration and are, therefore, primarily focused on by most data integration approaches in medicine [8-10].

This is also reflected in the DS category. In many cases, the storage requirements address the nature of data, availability of data, and data models from an interoperability point of view. These aspects are determined by not only the source system to be integrated into but also the data processing step. Thus, the latter can also be found in the data acquisition and processing categories. By contrast, the requirements in the categories MDM, DL, and DT seem underrepresented. With 3.2% (7/220) of requirements in the DT category, 2.3% (5/220) of requirements in DL, and 6.4% (14/220) in MDM, these categories are smaller than DA or DP. This leads us to the hypothesis that either our category scheme might differentiate too strongly for the implicit and explicit requirements given or that the processes of these categories are downstream of the acquisition and processing of data and thus have no direct relevance in the context of a technical concept or project phases. Other conceivable explanations might result from a general lack of research interest in DL-, DT-, or metadata-related requirements.

The same assumption can be applied to the DSC category. This category can also be considered a smaller category with 5.5% (12/220) of requirements, but these are with 18% (11/61) of publications. Thus, this category is characterized by a higher density of literature. The requirements in this category address security issues such as user access, logging of data access, or pseudonymization of patient data. The number of requirements allows us to conclude that either the authors are not aware of the need for DSC or there is no research interest in publishing these requirements in the context of medical data integration. Eventually, the challenges here might be concentrated either on partial aspects of DSC such as user authentication and pseudonymization or on other aspects that might have already been solved. It can also be assumed that DSC requirements are not included in publications in the context of the use case described here because they are perceived as self-evident or generally applicable.

Similarly, the requirements for data analytics are downstream processes when it comes to data integration. In this category too, the source density was low (14/61, 23% publications, to 20/220, 9.1% requirements). These requirements address individual analysis use cases such as clinical decision support based on the integrated data, the use of NLP methods for semantic annotation, or the extraction of data marts with a small intersection between publications. Although the requirements for the analysis of data to be integrated seem self-evident, they seem to play a minor role compared with the acquisition and processing of data. In our opinion, the specific analysis project might not have been sufficiently defined at the time the data integration was designed or implemented, or the downstream processes played a minor role according to the authors of the screened literature. At the same time, one can assume that DAS is conducted independently of data integration and, therefore, not discussed in a conceptual or technical context.

The MDM requirements, with 6.4% (14/220) of requirements covered by 25% (15/61) of identified publications, are consistent with the definition used. These can be clearly divided into 2 foci of requirements: effort reduction and improved accessibility. However, a similar pattern to that found with respect to DAS emerges relatively few articles concerning MDM were found in the literature. As the metadata are an auxiliary information to support integration, they might not be a mandatory requirement. This could also explain the small number of corresponding articles.

The result distribution clearly shows that the focus of integration projects in medical publications is on acquiring and processing data. Aspects such as storage, analysis, lineage, traceability, security, and MDM are of secondary importance and might, therefore, be addressed in the context of more clinical than technical or conceptual publications.

Another possible reason for the imbalance in the distribution among the categories could be the selection of stakeholders consulted by the authors of the articles. In many of the examined publications, it was not explicitly described which stakeholders contributed to implicit and explicit requirements for a data integration project and what expertise they had. However, only a comprehensive understanding of the needs of different stakeholders, such as interdisciplinary users, data providers, or administrators, can ensure a transparent development process by describing what a system should do and how it is expected to perform [20,24].

During this study, we collected the functional requirements of various data integration projects and developed a broad requirement catalog for the integration of medical data.

With this requirement catalog, we provide a tool to guide researchers and developers through the concept phase of the implementation of data integration processes and platforms. The requirement elicitation phase with different stakeholders is often characterized by the unawareness of one’s own requirements and by the lack of a common “vocabulary.” Here, the requirements of other projects can help support a common understanding and serve as the basis for further discussions.

In addition, the requirements of the catalog are suitable for developing a baseline for one’s own requirement process by identifying suitable requirements, transferring them to the requirement glossary, and adapting them to one’s own use case. In this context, it is worth mentioning that data integration projects are characterized by a comprehensive design phase compared with traditional software development [22].

To support the development phase, specific sprints as well as work packages and milestones can be derived on the basis of our requirements catalog. Beyond the concept phase, the catalog is also suitable for evaluating already implemented data integration processes against the requirements of other projects to support and improve one’s own systems.

Finally, not only does our catalog allow researchers to benefit from the requirements collected in other projects, but it also contributes to harmonized data integration in medicine and to the subsequent paradigm shift.

### Limitations

Although the review results clearly show that the search terms “requirements” and “data integration” are suitable to identify publications that contain corresponding functional requirements, there are a few considerations that limit the reach of our approach. First, it needs to be kept in mind that deriving functional requirements can be subject to selection bias because the process is based on a qualitative approach and is thus influenced by individual factors such as professional expertise and researchers’ perspectives, experiences, and research focuses.

Second, there is a risk of oversimplifying specific subaspects, especially when it comes to the elicitation and merging of multiple requirements from different publications into a single meaningful requirement. Haak et al [41], for instance, stated that “(a)ll types of DICOM data formats should be supported (including DICOM-convertible data) to avoid manual conversion steps before integration.” This statement has been incorporated into requirement *DA-02* by mentioning the inclusion of DICOM as another interoperability standard in the notes. We counteracted the risk of oversimplification by reconciling the derivation of requirements with 2 independent reviewers. Consequently, we do not claim general validity or acceptance of the presented results.

Researchers with other approaches might get different results owing to the nature of qualitative approaches, especially because implicit requirements are considered in addition to explicit ones, leaving room for interpretation. Although the *Results* section predominantly contains requirements with a large overlap among the publications analyzed, some requirements were also derived on the basis of individual mentions and may, therefore, only apply to the respective authors’ specific use case.

The third limitation is found in the adapted category scheme. In most cases, the examined publications focused on use case–specific aspects and did not provide a differentiated categorization of their requirements [51,52,57,66]. Therefore, in some cases, a granular differentiation between categories was not possible. For example, *DP-02* (“The system must process unstructured data by using natural language processing (NLP) methods.”) and *DAS-02* (“The system must analyze unstructured data by using natural language processing (NLP) methods.”) both address the need to process unstructured data using NLP methods. The focus of the DP requirement *DP-02* is on the use of NLP in an integrated procedure. By contrast, the DAS requirement *DAS-02* calls for the use of NLP in a downstream data analysis. Thus, some categories contain comparable requirements that could not be merged.

A similar disparity was observed in the MDM and DT categories. For example, the DT requirement *DT-05* refers to the need to be able to trace data back to its source. Source information is usually stored in metadata; therefore, this requirement creates an overlap between the two categories. This, in some cases, results in a lack of selectivity for the categorization of some requirements, leaving room for interpretation, as conducted by the reviewers. In ambiguous cases, the reviewers used the definitions established at the outset to minimize the bias of being influenced by one’s perspective.

However, the category scheme is considered state of the art and supported us in structuring and delineating the requirements.

Finally, we would like to point out that the compiled requirements catalog is not meant to be disjunctive; therefore, the requirements are not necessarily directly interrelated. This means that individual requirements may contradict each other. For example, *DA-05-1* (“The system must acquire data via a semantic integration layer based on source data information models.”) explicitly requires respect for the data model of a database of which data should be integrated from. By contrast, *DA-15* (“The system must acquire data independently of the source database’s structure*.*”) requires that data be integrated independently of the database structure. This is a direct contradiction because both requirements originate from different publications with different use cases.

Furthermore, the age of some publications is worth mentioning. For example, the oldest publication dates back to 2000. Accordingly, the individual aspects of the requirements may be outdated. However, we assume that older requirements might still be valid because of the following reasons: (1) in health care, there is a general resistance to replacing proven systems; (2) the system landscape in health care has grown historically, so even outdated legacy systems with limited interoperability capabilities continue to generate data that need to be integrated; (3) the translation of new technologies in health care is traditionally delayed owing to demanding regulations or legal concerns with respect to joint IT solutions [81]; and (4) functional requirements are meant to be technology agnostic.

Therefore, the users of the catalog are encouraged to identify and directly adopt requirements that intertwine and are relevant to their use case at hand. It may also be necessary to adapt these requirements. This applies particularly to the requirements that include examples in the notes.

### Comparison With Prior Work

The 61 publications we examined here directly or indirectly address data integration requirements, and they are first and foremost determined and motivated by a use case approach. Unlike our approach, other authors tend to explicitly mention requirements to address the project level of specific use cases or medical domains. Although Ayatollahi et al [51] elicited requirements to integrate genetic data into EHRs, Ganzinger et al [71] described requirements on the use case of data integration in a biomedical network, Daniel et al [52] reported on a core set of requirements for a semantic interoperability platform for cross-border semantic data integration, and Rance B et al [57] discussed the requirements for an integration project in the context of cancer research. Our approach is explicitly designed to go beyond single reaches and instead cover a cross-section of functional requirements at the level of generic data integration for any knowledge management environment. Furthermore, none of the publications examined formulated requirements in accordance with ISO 29148. Therefore, our work contributes to more structure and traceability at the level of individual requirements, in addition to providing a broad overview.

### Conclusions

The aim of this work was to offer a cross-section of functional data integration–related requirements defined in the existing literature. After a multistep procedure, we identified 220 distinct requirements from a total of 61 publications and arrived at the following conclusions for the domain of medical data integration.

In principle, scientific publications are a reliable source for identifying the functional requirements of medical data integration. Although the methodological approach of evaluating the state of research via a systematic literature search is not new, combining requirements engineering methods with a systematic literature review represents an innovative approach in the domain of medical data integration. By focusing on broad search terms, a large number of publications can be included.

Finally, a catalog of functional requirements is available from which scientists can extract relevant requirements for their projects. Building on this study, deriving a representative core set of requirements or a reference model for data integration will be evaluated in the future. Requirements with a large intersection among publications seem particularly suitable for this purpose.

## Appendix

Multimedia Appendix 1 Supplementary material. This appendix contains 3 worksheets. The worksheet “requirements” covers a list of the elicited functional requirements. The worksheet “articles” covers a list of the included articles. The worksheet “elicitation” covers the citations from the publications on which the surveyed requirements are based.

## Abbreviations

DA: data acquisition
DAS: data analysis
DICOM: Digital Imaging and Communications in Medicine
DL: data lineage
DP: data processing
DS: data storage
DSC: data security
DT: data traceability
EHR: electronic health record
MDM: metadata management
NLP: natural language processing
PRISMA: Preferred Reporting Items for Systematic Reviews and Meta-Analyses
